# pAKT pathway activation is associated with *PIK3CA* mutations and good prognosis in luminal breast cancer in contrast to p-mTOR pathway activation

**DOI:** 10.1038/s41523-019-0102-1

**Published:** 2019-01-31

**Authors:** Amir Sonnenblick, David Venet, Sylvain Brohée, Noam Pondé, Christos Sotiriou

**Affiliations:** 1 Oncology Division, Tel Aviv Sourasky Medical Center, and Sackler Faculty of Medicine, Tel Aviv University, Tel Aviv, Israel; 2 Breast Cancer Translational Research Laboratory, Institut Jules Bordet, Université Libre de Bruxelles, Brussels, Belgium

## Abstract

Numerous studies have focused on the PI3K/AKT/mTOR pathway in estrogen receptor positive (ER) breast cancer (BC), as a linear signal transduction pathway and reported its association with worse clinical outcomes. We developed gene signatures that reflect the level of expression of phosphorylated-Serine473-AKT (pAKT) and phosphorylated-Serine2448-mTOR (p-mTOR) separately, capturing their corresponding level of pathway activation. Our analysis revealed that the pAKT pathway activation was associated with luminal A BC while the p-mTOR pathway activation was more associated with luminal B BC (Kruskal–Wallis test *p* < 10^−10^). pAKT pathway activation was significantly associated with better outcomes (multivariable HR, 0.79; 95%CI, 0.74–0.85; *p* = 2.5 × 10^−10^) and *PIK3CA* mutations (*p* = 0.0001) whereas p-mTOR pathway activation showed worse outcomes (multivariable HR,1.1; 95%CI, 1.1–1.2; *p* = 9.9 × 10^−4^) and associated with *p53* mutations (*p* = 0.04). in conclusion, our data show that pAKT and p-mTOR pathway activation have differing impact on prognosis and suggest that they are not linearly connected in luminal breast cancers.

## Molecular signaling: Two components of same pathway have divergent effects

One of the key signaling pathways involved in regulating cancer cell growth and survival is not linearly connected in luminal breast cancers, as researchers had long assumed. A team led by Amir Sonnenblick from Tel Aviv University, Israel, and Christos Sotiriou from Institut Jules Bordet, Belgium, studied tumors with elevated expression of two different proteins in the PI3K/AKT/mTOR pathway. They showed that tumors with activated AKT had distinct gene expression profiles from tumors with activated mTOR. What’s more, patients with AKT activation had better outcomes on average and were more likely to carry mutations in the oncogene PIK3CA, whereas patients with mTOR activation tended to relapse sooner and were more likely to carry mutations in the tumor suppressor p53. The findings suggest that doctors should look closely at PI3K/AKT/mTOR signaling activation when personalizing treatment decisions.

## Introduction

The phosphatidylinositol 3-kinase (PI3K)/AKT/mTOR-signaling pathway mediates key cellular functions, including growth, proliferation, and survival and is frequently involved in carcinogenesis, tumor progression, and metastases.^1^ Numerous studies have focused on the PI3K/AKT/mTOR pathway in estrogen receptor positive (ER-positive) breast cancer (BC) and have shown that *PIK3CA* mutations are frequent, that the PI3K/AKT/mTOR-signaling pathway is often dysregulated and that both correlate with worse clinical outcomes.^2–4^ As a consequence, a large number of drugs targeting the various components of this pathway have been developed.^5^ Everolimus (an mTOR inhibitor) is currently the only approved drug targeting mTOR based on the results of the BOLERO-2 trial.^6^

While AKT is activated by phospholipid binding and activation loop phosphorylation at Threonine308 by PDK1 and by phosphorylation within the carboxy terminus at Serine473, mTOR is phosphorylated at Serine2448 via the PI3K-signaling pathway.^7^ AKT activates the mTOR complex 1 (mTORC1) which in addition to mTOR contains mLST8, PRAS40, and RAPTOR.^8^ This activation involves phosphorylation of tuberous sclerosis complex 2 (TSC2), which blocks the ability of TSC2 to act as a GTPase-activating protein, thereby allowing accumulation of Rheb-GTP and mTORC1 activation. AKT can also activate mTORC1 by PRAS40 phosphorylation, thereby relieving the PRAS40-mediated inhibition of mTORC1.^9^

The PI3K/AKT/mTOR pathway is usually considered as a linear signal transduction pathway in BC, however in the ER-positive disease, we have previously shown that *PIK3CA* mutations were associated with relatively low mTORC1 functional output and with good outcomes in patients who received adjuvant tamoxifen monotherapy.^4^ Therefore, to gain better insight into the relative contribution of each of the signaling pathways which lie downstream to PI3K (namely AKT and mTOR) to BC outcomes, we have developed a novel in silico approach which assessed the activation of each of these signaling pathways separately, by integrating reverse phase protein array (RPPA) and matched gene expression.

## Results

### pAKT pathway activated and p-mTOR pathway activated ER-positive early BCs are associated with distinct and exclusive gene expression profiles

**Fig. 1:**
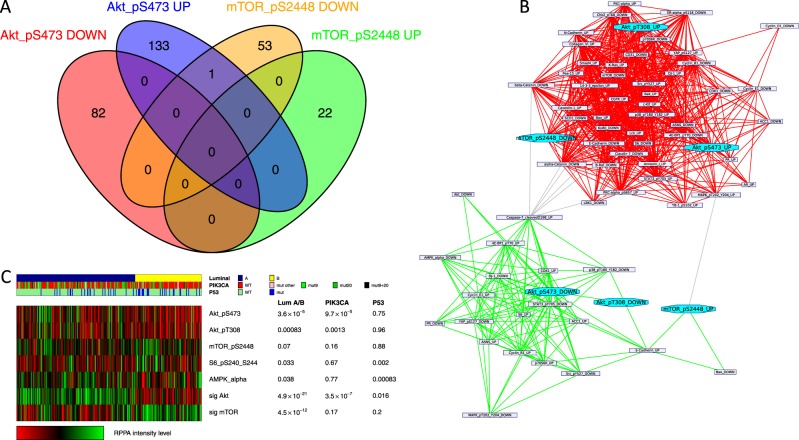
pAKT and p-mTOR signatures derived from the TCGA. **a** Venn diagram shows no intersection between the pAKT and p-mTOR gene signatures. **b** Network representation of the gene signatures. Each node represents the genes up-regulated or down-regulated in the signature. Edges show signatures sharing a significant number of genes. Network clustering shows the tendency of these signatures to cluster together according to their proliferation status. **c** Integrated analysis of the *PIK3CA*/pAKT/m-TOR pathway in the TCGA. Luminal breast cancer subtypes differ by pAKT and p-mTOR activity. The panel includes a protein-based (RPPA) proteomic status. Tumors were ordered first by mRNA subtype (luminal A versus B). *P* values were calculated using the Mann–Whitney test

We first derived two distinct signatures whose expression levels could predict AKT and mTOR pathway activation through pAKT and p-mTOR RPPA levels by computing the differentially expressed genes between tumor samples with high and low RPPA levels of pAKT (respectively, activated and inactivated AKT pathway) and p-mTOR proteins (respectively, activated and inactivated mTOR pathway), using ER-positive tumors from the TCGA repository. It is important to note that the two signatures did not share any common genes (Fig. 1a). We next sought to assess their biological and clinical relevance in BC. Firstly, we compared both signatures to the reference classes of the Gene Ontology and the mSigDB signatures repositories using the Broad Institute site.^10^ This showed that the pAKT signature was significantly enriched in genes up-regulated in less aggressive invasive BC tumors (e.g. grade 1 vs. grade 3^11^; fdr = 2 × 10^−27^). In contrast, the p-mTOR signature was enriched in genes expressed in mammary stem cells and more aggressive luminal B cancers^11,12^ (fdr = 2 × 10^−7^, fdr = 3 × 10^−5^, respectively). A network clustering analysis using the pAKT and p-mTOR signatures, as well as other RPPA-derived signatures that demonstrated a significant intersection with them, identified two main sub-networks according mainly to their proliferation status, namely pAKT-high/p-mTOR-low and pAKT-low/p-mTOR high characterized with low and high proliferation levels, respectively (Fig. 1b). These observations were confirmed when analyzing the TCGA RPPA dataset (Figs. 1c and S1). AKT pathway was more often activated (elevated pAKT expression) in luminal A cancers whereas mTOR pathway was more often activated (elevated p-mTOR and pS6, an mTOR downstream target), in luminal B subtypes (Figs. 1c and S2). Next, we sought to determine how the pAKT and p-mTOR signatures correlate with other signatures and RPPA markers of the pathway. As shown in Fig. S3, the pAKT signature negatively correlates with downstream effectors of the pathway while the p-mTOR signature positively correlates with them.

Altogether, these results demonstrate that the pAKT and p-mTOR pathways, assessed through these RPPA-based gene expression signatures, have exclusive distribution according to luminal molecular subtypes and are not necessarily linearly connected.

### Association of the pAKT and p-mTOR pathway activation with clinical outcome in patients with ER-positive early BC

**Fig. 2:**
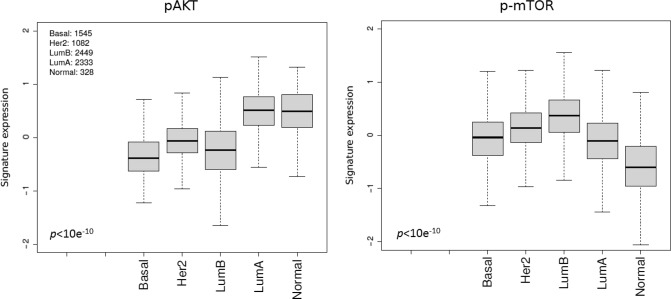
pAKT (right) and p-mTOR (left) gene signatures expression in publicly available microarray datasets according to the PAM50 breast cancer subtype. Kruskal–Wallis *p-*value is shown

**Fig. 3:**
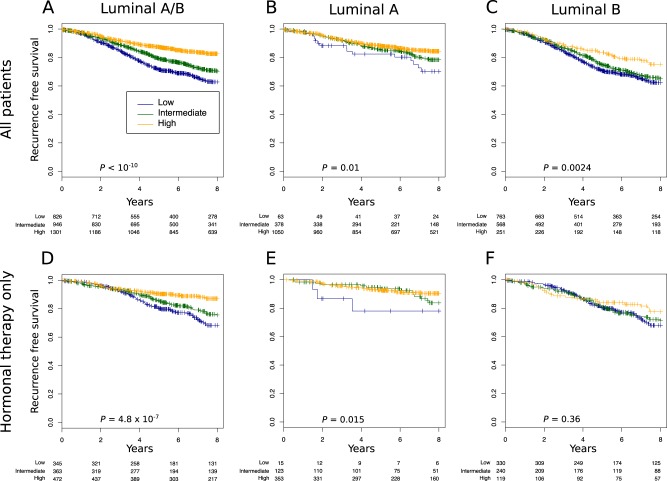
High pAKT gene signature expression is associated with good prognosis in the luminal subtype. **a**–**f** We assessed the prognostic value of tertiles of pAKT gene signature expression in: **a** all luminal patients treated or not treated (*n* = 3073), **b** luminal A (*n* = 1491), **c** Luminal B (*n* = 1582), **d** all luminal treated with only hormonal therapy (*n* = 1180), **e** luminal A treated with only hormonal therapy (*n* = 491), and **f** luminal B treated with only hormonal therapy (*n* = 689). Significance (*p*-value) of differences in survival between patient groups defined by tertiles of pAKT signature expression is estimated by log-rank test. The analysis presented includes patients with lymph node-negative and lymph node-positive cancers

**Fig. 4:**
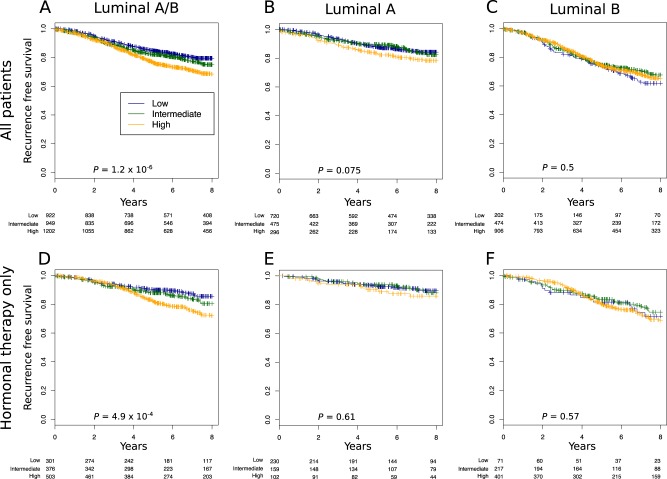
High p-mTOR gene signature expression is associated with bad prognosis in the luminal subtype. **a**–**f** We assessed the prognostic value of tertiles of p-mTOR gene signature expression in: **a** all luminal patients treated or not treated (*n* = 3073), **b** luminal A (*n* = 1491), **c** Luminal B (*n* = 1582), **d** all luminal treated with only hormonal therapy (*n* = 1180), **e** luminal A treated with only hormonal therapy (*n* = 491), and **f** luminal B treated with only hormonal therapy (*n* = 689). Significance (*p* values) of differences in survival between patient groups defined by tertiles of p-mTOR signature expression is estimated by log-rank test. The analysis presented includes patients with lymph node-negative and lymph node-positive cancers

**Fig. 5:**
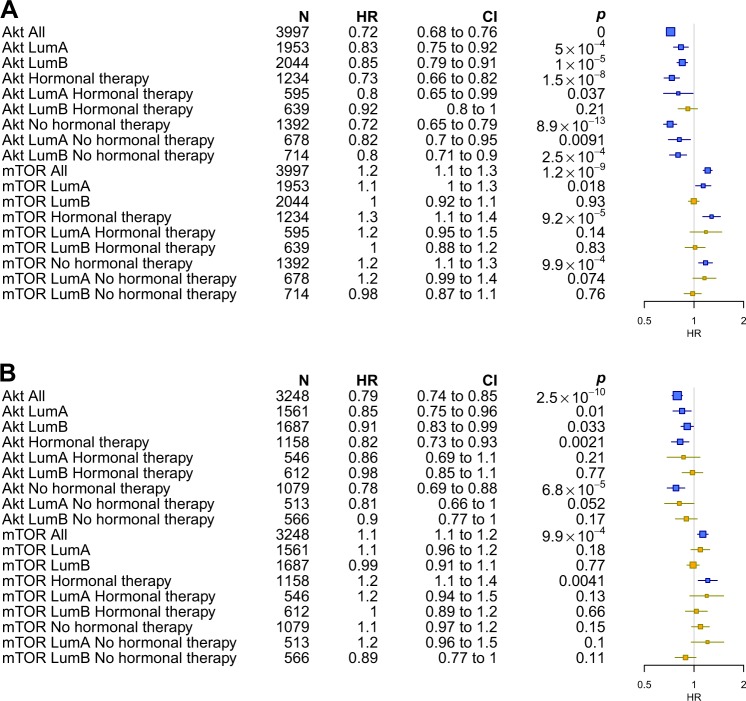
Forest plots showing the hazard ratios of the recurrence free survival of pAKT and p-mTOR gene signatures treated as a continuous variable using Cox univariate **a** and multivariable analysis **b**, in the pooled analysis. For multivariate analysis, we considered the following variables: age, tumor size, grade, and nodal status. Signatures with nominal significant effect (*p* < 0.05) are shown in blue

To ascertain the impact of each pathway on outcomes in ER-positive BC, we applied the pAKT and p-mTOR signatures on a dataset composed of 38 publicly available microarray datasets. We first assessed whether pAKT or p-mTOR pathway activation were associated with any particular luminal subtype. As expected, in the pooled set analysis pAKT pathway activation was significantly associated will luminal A cancers (*p* < 10^−10^) whereas p-mTOR pathway activation was associated with luminal B cancers (*p* < 10^−10^) (Fig. 2). We next assessed whether pAKT and p-mTOR pathway activation were correlated with outcomes (RFS) in ER-positive patients with relapse data available. As shown in Figs. 3 and 5, pAKT pathway activation was significantly associated with better outcomes in all luminal patients (multivariable HR, 0.79; 95% CI, 0.74–0.85; *p* = 2.5 × 10^−10^). Similar results were obtained with a dataset consisting of patients treated with endocrine therapy only (multivariable HR, 0.82; 95% CI, 0.73–0.93; *p* = 0.002). Indeed, patients with pAKT pathway activation had better outcomes irrespective of their specific subtype (luminal A multivariable HR, 0.85; 95% CI, 0.75–0.96; *p* = 0.01; luminal B HR, 0.91; 95% CI, 0.83–0.99; *p* = 0.033). In contrast, patients with p-mTOR pathway activation had significantly worse outcomes in all luminal patients (multivariable HR, 1.1; 95% CI, 1.1–1.2; *p* = 9.9 × 10^−4^) and this remained true when tested in the dataset consisting of patients treated with endocrine therapy only (multivariable HR, 1.2; 95% CI, 1.1–1.4; *p* = 0.004) (Figs. 4 and 5).

**Fig. 6:**
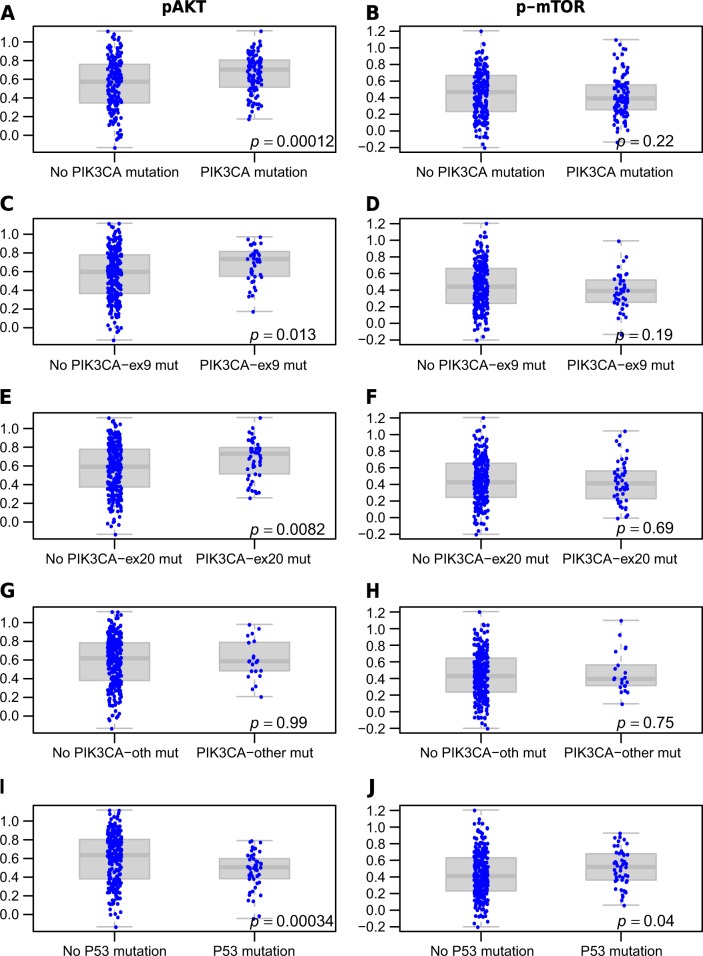
Expression of pAKT (**a**, **c**, **e**, **g**, **i**) and p-mTOR (**b–d, f**, **h**, **j**) gene signatures levels in *PIK3CA* (all, exon9, exon20 or others) and *P53* mutated and wild type samples in an independent RNA sequencing set (*n* = 309)

Next, we assessed the association between the *PIK3CA* and *P53* mutation status and pAKT and p-mTOR pathway activation in an independent set, namely the TCGA BC patients with RNA-sequence gene expression data that were not used to design the signatures. While the pAKT signature was associated with *PIK3CA* mutations (*p* = 0.0001), the p-mTOR signature was not (*p* = 0.22) (Fig. 6a, b). The opposite was true for *P53* mutations, which were positively correlated with p-mTOR pathway activation (*p* = 0.04), and negatively correlated with pAKT (*p* = 0.0003) (Fig. 6i, j). Analysis of the *PIK3CA* mutations by exon led to similar results (Fig. 6), although mutations outside of exons 9 and 20 seemed less associated with pAKT pathway activation.

Finally, in an effort to identify whether these signatures could predicts response to mTOR inhibitors, we evaluated another data set of neo-adjuvant patients treated with Everolimus.^13^ Analysis of the correlations between the effectiveness of this treatment and the developed signatures suggests as expected that the pAKT signature is associated with less response to Everolimus (*r* = 0.45; *p* = 0.031, Fig. S4).

Overall, our data suggests that pAKT and p-mTOR pathway activation as assessed through the respective signatures, despite being major components of the same overarching pathway (PI3K), have distinctly different impacts on disease biology and consequently on outcomes in early disease.

## Discussion

The goal of the present study was to better understand the distinct contribution to disease biology and clinical outcomes of signaling through the AKT and mTOR downstream pathways, which typically occur as part of PI3K pathway activation in luminal BCs. We found that pAKT and p-mTOR were differentially expressed according to luminal subtypes, implying different degrees of pathway activation, and that, more importantly, the pathways were not linearly connected. Additionally, we found that pAKT pathway activation was positively associated with *PIK3CA* mutations whereas the opposite was observed with p-mTOR pathway activation. In contrast, pAKT pathway activation was associated with good clinical outcome despite its known tumorigenic effects. Between 30% and 40% of BCs, especially ER-positive tumors, have mutations in *PIK3CA*.^14^ The vast majority of the *PIK3CA* mutations are missense mutations which are positioned in the helical domain (exon 9, mostly: E545K and E542K) and the kinase domain (exon 20, mostly H1047R) in hotspot clusters.^15^ These mutations have direct effect on AKT phosphorylation. The effect of *PIK3CA* mutations/pAKT on prognosis is mixed in early BC.^16^ We found that exons 9 and 20 mutations in *PIK3CA* were more associated with pAKT than mutations in other exons.

We previously reported that *PIK3CA* mutations were associated with improved outcome and low levels of signaling through the mTOR pathway in BC.^4,17^ Several possible hypotheses were raised regarding the reasons for this. Some data available on PP2A and PML, both known to have an inhibitory effect on both AKT and mTOR,^18,19^ have suggested that they may be upregulated in *PIK3CA-*activated tumors. Negative feedback regulation in PI3K-mediated cells through the insulin receptor substrate^20^ and relatively weak pathway activation in *PIK3CA*-mutated cancers have also been suggested as possible explanations for low levels of signaling through mTOR in ER-positive BC.

According to our findings only pAKT pathway activation was found to be significantly different between the luminal subtypes (A and B) and *PIK3CA* wildtype versus mutant, whereas p-mTOR pathway activation was not significant for both. The inconclusive and relative activation of p-mTOR by the mutant *PIK3CA* may be also attributed to the different roles and activators of mTOR and the fact that mTOR is at the cross section of multiple signaling pathways. Several studies have clearly demonstrated that mTOR is a direct substrate for the AKT kinase and identified Serine2448 as the AKT target site in mTOR.^21^ However, additional studies have demonstrated that rapamycin, an inhibitor of mTOR function, blocks serum-stimulated Serine2448 phosphorylation of mTOR in an AKT-independent manner and identified S6 kinase as a major effector of mTOR phosphorylation at Serine2448.^22^ Indeed, our analysis of the TCGA data shows that the S6 kinase (downstream of mTOR) is associated with luminal B and *P53* mutations suggesting that while mTOR itself is at the cross section of conflicting pathways its downstream targets are not *PIK3CA* dependent. In addition, there are alternative kinases that can activate the mTOR pathway independently of AKT, such as RSK which leads to phosphorylation of TSC resulting in increased mTOR signaling and the PDK1–SGK1 axis that can sustain mTOR activity upon AKT suppression.^23–25^

The primary TCGA report, which investigated all BC subtypes,^26^ confirmed a high frequency of *PIK3CA* mutations in luminal BC. Multiple platforms, which examined the relationship between *PIK3CA* mutation and protein expression, have demonstrated that pAKT and pS6 were not elevated in *PIK3CA*-mutated luminal cancers; instead, they were highly expressed in basal-like and HER2 subtypes. Our dataset, which is restricted to luminal cancers, demonstrated that pAKT pathway activation is associated with luminal A/*PIK3CA* mutations and good prognosis, while p-mTOR/pS6 is not, suggesting that the presence of HER2 and basal subtype in the primary analysis of the TCGA masked these observations.

In conclusion our data suggest that the AKT and mTOR pathways are not linearly connected in luminal BCs. pAKT pathway activation is associated with *PIK3CA* mutations, luminal A and good prognosis, while p-mTOR pathway activation is associated with luminal B, *P53* mutations, and bad prognosis. These results may have important clinical implications considering that in low p-mTOR BCs, treatment with mTOR inhibitors, such as everolimus, which is highly toxic, will possibly be of lower value since the pathway is not activated. Additionally, pAKT pathway activation, as measured through our gene signature, can add to presently used outcome prediction tools in both luminal A and luminal B tumors.

## Methods

### Computation of RPPAs-based signatures

We downloaded clinic-pathological, normalized gene expression and RPPA data from the publicly available TCGA repository using its online bioinformatics tools^26^ (Fig. S1 flow chart). ER-positive early BCs were analyzed based on the RPPA proteomic levels. 265 samples with available gene expression and RPPA data were considered as luminal (166 Luminal A and 99 Luminal B according to PAM50 computed on the cBioPortal website^27^). To identify the genes that were differentially expressed between the low and high expression groups, and to find the genes that would optimize the predictive power of our signatures, we used a machine learning approach as previously described.^28^ After this process, we were left with 69 signatures (Supplementary Data) presenting a relevant AUC for proteomic status prediction. Among others, p-mTOR achieved an AUC of 0.71 (*p* ~ 10^−6^) and pAKT an AUC of 0.77 (*p* ~ 10^−11^) in both luminal A and B cancers.

### Code availability

The expression levels of the signatures in the gene expression datasets were computed as previously described.^28^ In brief, we evaluated using a nested 10-fold cross validation the maximal Benjamini–Hochberg false discovery rate and the minimal gene fold change that would optimize the ability of the differentially expressed genes to predict the high/low status of the RPPA in luminal A and B patients together and separately. While the parameters were selected in a 10-fold cross validation, the procedure itself was assessed using a nested cross validation. All analyses were performed using the genefu package of the R (v3.2)/bioconductor (v1.18) statistical suite.

### Network analysis and clustering

Network analysis and clustering was performed as previously described.^28^ The MCL graph clustering algorithm^29^ was applied. Visualization of the network has been rendered using the yED software.

### Gene-expression data and statistical analyses

We analyzed 38 gene expression datasets totaling more than 7000 tumors (detailed in Table 1). To ensure comparability of expression values across multiple data sets, a 0.95 quantile normalization was performed. Differences in expression of pAKT and p-mTOR signatures according to subtype were examined using the Kruskal–Wallis test. Survival outcome data are presented as recurrence free survival (RFS). Survival plots according to the pAKT and p-mTOR signatures tertiles were drawn using the Kaplan–Meier method. Association of the signatures (i.e. pathway activation) with good or bad outcomes were computed using uni-variate or multi-variate Cox regression analyses and data were presented as forest plots. For multivariate analysis, we considered the following variables: age, tumor size, grade, and nodal status. To assess the correlation between the *PIK3CA* mutation status and AKT and p-mTOR gene pathway activation, as represented by the gene signature scores, we analyzed the TCGA cohort of RNA sequenced data that was not used for the computation of the signatures (309 samples), and for which both mutational and gene expression data were available. Each sample was considered as mutated or not (so a sample with four mutations was considered just like a sample with one mutation). All mutations were taken into account. *PIK3CA* mutations were also analyzed by specific exons (exons 9, 20, and all others grouped together).

**Table 1:** The sources and locations for the 38 gene expression datasets analyzed

Dataset	No. of patients	Permanent identifier	References
NKI	337	10.1038/415530a	^31, 32^
UCSF	162	GSE123833	^33, 34^
STNO2	122	GSE4335	^35^
NCI	99	10.1073/pnas.1732912100	^36^
UNC4	337	GSE18229	^37^
CAL	118	E-TABM-158	^38^
MDA4	129 (65)^‡^	GSE123832	^39, 40^
KOO	88	GSE123831	^41^
HLP	53	E-TABM-543	^42^
EXPO	353	GSE2109	^43^
VDX	344	GSE2034/GSE5327	^44, 45^
MSK	99	GSE2603	^46^
UPP	251 (190)^‡^	GSE3494	^47^
STK	159	GSE1456	^48^
UNT	137 (92)^‡^	GSE2990	^36, 49^
DUKE	171	GSE3143	^50^
TRANSBIG	198	GSE7390	^51^
DUKE2	160	GSE6961	^52^
MAINZ	200	GSE11121	^53^
LUND2	105	GSE5325	^54^
LUND	143	GSE5325	^55^
FNCLCC	150	GSE7017	^56^
EMC2	204	GSE12276	^57^
MUG	152	GSE10510	^58^
NCCS	183	GSE5364	^59^
MCCC	75	GSE19177	^60^
EORTC10994	49	GSE1561	^61^
DFHCC	115	GSE19615	^62^
DFHCC2	84 (75)^‡^	GSE18864	^63^
DFHCC3	40 (26)^‡^	GSE3744	^64^
DFHCC4	129	GSE5460	^65^
MAQC2	230	GSE20194	^66^
TAM	345 (242)^‡^	GSE6532/GSE9195	^67^
MDA5	298	GSE17705	^68^
VDX3	136	GSE12093	^69^
PNC	248	GSE20713	^70^
TCGA	517	https://tcga-data.nci.nih.gov/docs/publications/brca_2012/	^26^
METABRIC	1643	EGAS00000000083	^14^

^‡^Duplicated patients were removed from few datasets for the estimation of concordance and prognostic value

### Reporting summary

Further information on experimental design is available in the Nature Research Reporting Summary linked to this article.

## Supplementary information

SUPPLEMENTAL MATERIAL

Reporting Summary

Supplementary Data

## Data Availability

The sources and locations for the 38 gene expression datasets analyzed during the current study are available in Table 1 and the figshare repository 10.6084/m9.figshare.7461776.^30^

## Supplementary information

**Supplementary information** accompanies the paper on the *npj Breast Cancer* website (10.1038/s41523-019-0102-1).
