# Supplementary material for: pAKT pathway activation is associated with PIK3CA mutations and good prognosis in luminal breast cancer in contrast to p-mTOR pathway activation
Source: NPJ Breast Cancer. 2019 Jan 31;5:7. doi: 10.1038/s41523-019-0102-1 (PMC6355773; doi:10.1038/s41523-019-0102-1)
Supplement: Supplementary file 1 — SUPPLEMENTAL MATERIAL [file 41523_2019_102_MOESM1_ESM.pdf]

Supplementary information

Figure S1

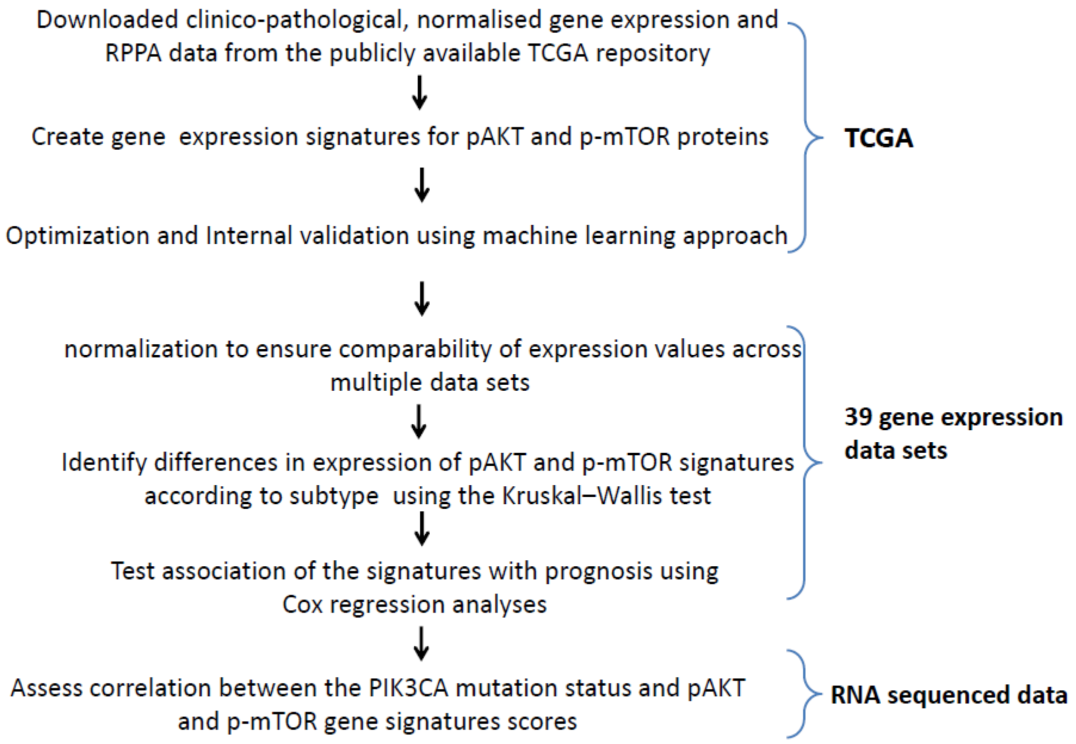

Figure S1. Flow chart – pipeline undertaken for the analysis.

Figure S2

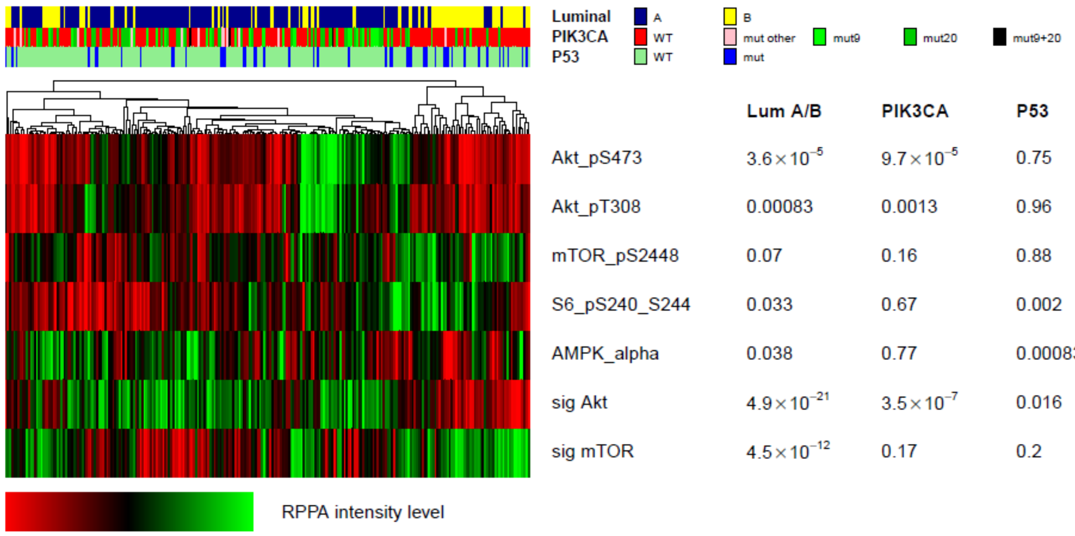

	Lum A/B	PIK3CA	P53
Akt_pS473	3.6×10^{-5}	9.7×10^{-5}	0.75
Akt_pT308	0.00083	0.0013	0.96
mTOR_pS2448	0.07	0.16	0.88
S6_pS240_S244	0.033	0.67	0.002
AMPK_alpha	0.038	0.77	0.00083
sig Akt	4.9×10^{-21}	3.5×10^{-7}	0.016
sig mTOR	4.5×10^{-12}	0.17	0.2

Figure S2. Unsupervised clustering of the PIK3CA/pAKT/m-TOR pathways in the luminal cancers TCGA. Luminal breast cancer subtypes differ by pAKT and p-mTOR activity (RPPA and expression signatures). P values were calculated using the Mann Witney test.

Figure S3

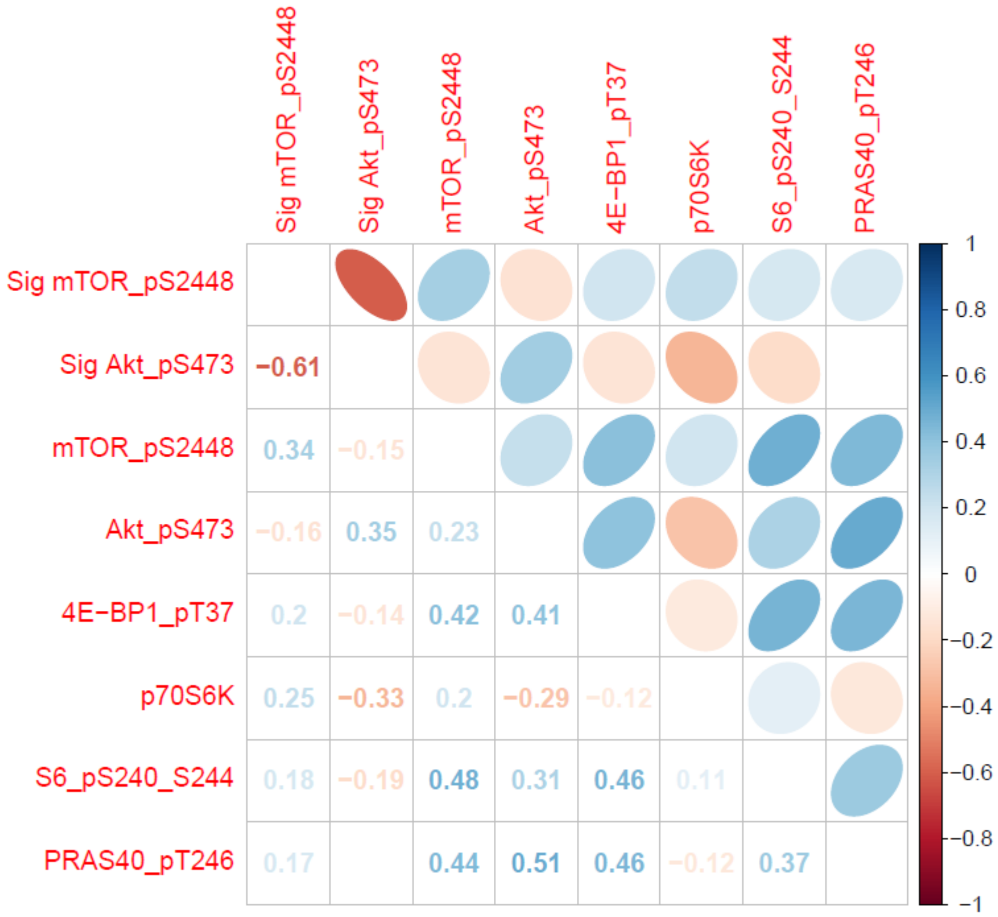

Figure S3: A graphical display of a correlation matrix that shows how the pAKT and p-mTOR signatures correlate with other signatures and RPPA markers of the pathway. Positive correlations are displayed in blue and negative correlations in red color. Color intensity and the direction of the circle are proportional to the correlation coefficients. In the right side of the correlogram, the legend color shows the correlation coefficients and the corresponding colors.

Figure S4

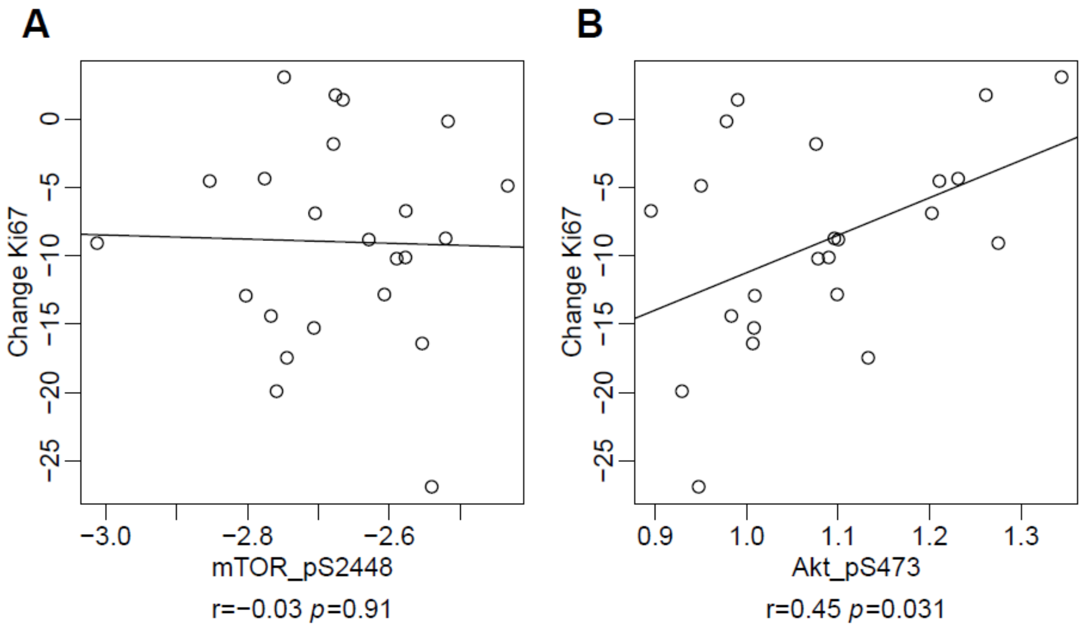

Figure S4. Correlation between p-mTOR (A) and pAKT (B) gene signatures levels in everolimus pre-treated samples according to tumor response based on Ki67% change in an independent RNA expression set (N=23).
